# Stat3 Expression and Its Correlation with Proliferation and Apoptosis/Autophagy in Gliomas

**DOI:** 10.1155/2008/219241

**Published:** 2009-04-27

**Authors:** Valentina Caldera, Marta Mellai, Laura Annovazzi, Guido Valente, Luciana Tessitore, Davide Schiffer

**Affiliations:** ^1^Neuro-bio-oncology Center, Policlinico di Monza Foundation, University of Turin, Via Pietro Micca, 29, 13100 Vercelli, Italy; ^2^Clinical and Experimental Medical Department, University of East Piedmont, 28100 Novara, Italy; ^3^Department of Chemical, Food, Pharmaceutical and Pharmacological Sciences (DISCAFF), University of East Piedmont, 28100 Novara, Italy

## Abstract

Signal transducer and activator of transcription-3 (Stat3) was studied along with several steps of the PI3/Akt pathway in a series of 64 gliomas that included both malignant and low-grade tumors, using quantitative immunohistochemistry, Western blotting, and molecular biology techniques. The goal of the study was to investigate whether activated Stat3 (phospho-Stat3) levels correlated with cell proliferation, apoptosis, and autophagy. Stat3 and activated Akt (phospho-Akt) expression increased with malignancy grade, but did not correlate with proliferation and survival within the category of glioblastomas. A correlation of Stat3 with Akt was found, indicating a regulation of the former by the PI3/Akt pathway, which, in turn, was in relation with EGFR amplification. Stat3 and Akt did not show any correlation with apoptosis, whereas they showed an inverse correlation with Beclin 1, a stimulator of autophagy, which was rarely positive in glioblastomas. Autophagy seems then to be inactivated in malignant gliomas.

## 1. Introduction

Stat3 is a member of a family of latent transcription factors that transduce extra-cellular signals, such as those mediated by cytokines and growth factors [1]. Stat3 is activated by the phosphorylation of a tyrosine residue (phospho-Stat3), which leads to its dimerization and nuclear translocation. It transcriptionally regulates the expression of genes responsible for proliferation and survival. pStat3 has been shown to suppress apoptosis in many cancers [2, 3].

pStat3 is ubiquitously expressed in mammalian cells and is constitutively activated in glioblastomas by IL-6 [4]. It has been shown that in many cancers when pStat3 is inhibited, its target genes, Bcl-X_L_, c-myc, and Cyclin D1, are down-regulated, and cells undergo apoptosis [3, 5, 6].

pStat3 is not detected in normal brain tissue. It is activated by aberrant EGFR signaling and IL-6 in malignant gliomas [7], and it may be a rational therapeutic target; WP10066, similar to AG490, has been shown to inhibit pStat3 and to induce apoptosis in vitro and in vivo [8]. A pStat3 mutant can induce cellular transformation and tumor formation in nude mice by binding DNA and activating transcription [5], and inhibition of pStat3 may promote the efficacy of immunotherapy [9]. In a series of diffuse gliomas, pStat3 has been found to be focally expressed in less than 9% of gliomas [10]. However, in another series, it was highly expressed at almost equal levels in anaplastic astrocytomas and glioblastomas (55.6% versus 56.4%), together with pAkt, and its expression also correlated with EGFR status (EGFRvIII), but had no predictive value [11]. pStat3 was found to be positive in 40% of gliomas and correlated with histological grades [12]. Recently, it has been demonstrated that both inhibitors of the PI3/Akt pathway [13] and pStat3 can induce autophagy in glioma cells [14].

Autophagy is a caspase-independent process of degradation, characterized by the formation of autophagosomes and their fusion with lysosomes [15]. It is regulated by pmTOR and its complexes [16], by autophagy genes (ATG) and Beclin 1 [17]. Beclin 1 is also known as Atg6, and is a component of a complex that includes the class III phosphatidylinositol-3-kinase and is an autophagic stimulator [16].

Quantitative evaluation of pStat3 expression in gliomas is difficult due to the regional heterogeneity of its expression. We investigated pStat3 expression and its correlations with histological grade, EGFR aberrations, PTEN mutations, pAkt expression, cell proliferation, and apoptosis/autophagy in a series of gliomas.

## 2. Materials and Methods

Surgical samples were collected from the Department of Neuroscience, University of Turin, and from the Clinical and Experimental Medical Department of East Piedmont, University of Novara. Sixty four gliomas were studied: 34 glioblastomas (GBMs), 10 grade III anaplastic astrocytomas, 10 grade II astrocytomas, and 10 oligodendrogliomas (5 grade II and 5 grade III), diagnosed according to the WHO.

All the tumor samples were from first surgery with partial or total removal. No recurrence and no second-operation materials were studied.

Surgical samples were fixed in buffered formalin, embedded in paraffin and cut in 5-micron thick serial sections.

### 2.1. DNA Extraction

Genomic DNA was extracted from the 34 formalin-fixed, paraffin-embedded GBM samples according to a standard phenol-chlorophorm protocol. Only proliferating areas were selected.

### 2.2. EGFR (Epidermal Growth Factor Receptor) Amplification

EGFR amplification status was assessed as previously described [18]. Fluorescent PCR products were analyzed by capillary electrophoresis on an ABI PRISM 3100 Genetic Analyzer (Applied Biosystems, Foster City, CA, USA). The amplification status of the EGFR gene was determined by measuring the EGFR/INF-*γ* ratio: a ratio >2.09 was taken as evidence of more than two copies of the EGFR gene.

### 2.3. PTEN (Phosphatase and Tensin Homolog) Mutation Analysis

The PTEN gene was amplified as ten polymerase chain reaction (PCR) fragments covering the 9 exons and at least 50 bp of flanking intronic sequence.

All the fragments were amplified using the same protocol; an initial denaturation at 96°C for 10 minutes followed by 94°C for 30 seconds, 58°C for 30 seconds, and 72°C for 30 seconds, for 33 cycles. A final elongation step of 10 minutes at 72°C was added. The reactions were performed in a total volume of 25 *μ*L containing 50 mM KCl, 10 mM Tris-HCl (pH 8.3), 1.5 mM MgCl_2_, 250 *μ*M of each dNTP, 1 unit of Taq Gold polymerase (Applied Biosystems), 10 pmol of each primer and 100 ng of genomic DNA. Primer sequences are available upon request.

Prior to sequencing, unincorporated dNTPs and primers were removed by using MultiScreen PCR filter plates on a MultiScreenHTS vacuum manifold (Millipore, MA Billerica, USA).

Each PCR product was further analyzed by direct DNA sequencing in both directions on an ABI PRISM 3100 Genetic Analyzer using the BigDye Terminator version 1.1 cycle sequencing Kit.

### 2.4. Immunohistochemistry (IHC)

The following primary antibodies were used. Mouse monoclonal antiphospho-Stat3 (Tyr705), diluted 1:40 (*#*9138, Cell Signaling Technology, Beverly, MA, USA); mouse monoclonal antiphospho-Akt (Ser473), diluted 1:100 (*#*4051, Cell Signaling Technology); rabbit polyclonal anti-EGFRwt, diluted 1:50 (*#*2232, Cell Signaling Technology); rabbit polyclonal anti-EGFRvIII, diluted 1:50 (SC1031, GenScript Corporation, Piscataway, NJ, USA); mouse monoclonal anti-PTEN (A2B1), diluted 1:1000 (sc-7974, Santa Cruz Biotechnology, Santa Cruz, CA, USA); mouse monoclonal anti-Ki-67/MIB.1, diluted 1:100 (M7240, Dako, Carpinteria, CA, USA); rabbit polyclonal anti-Caspase-3, diluted 1:20 (AB3623, Chemicon International Inc., Temecula, CA, USA); rabbit polyclonal anti-PARP1, diluted 1:200 (*#*9542, Cell Signaling Technology); rabbit polyclonal anticleaved PARP1 (Asp214), diluted 1:50 (*#*9541, Cell Signaling Technology), rabbit polyclonal anti-Beclin 1, diluted 1:200 (sc-11417, Santa Cruz Biotechnology).

Besides the H&E method, immunohistochemistry was performed on consecutive sections using a standard streptavidin-biotin system (Dako) with diaminobenzidine as the substrate (DAB, Roche Diagnostics GmbH, Penzberg, Germany), and the sections were counterstained with Harris' hematoxylin. Antigen retrieval was performed by microwaving the sections in 0.01 M citrate buffer (pH 6.0 or 7.4) (3 × 3 minutes at 600 W). Negative controls were only incubated with the secondary antibody. The positive controls were human breast cancer samples for pStat3 and pAkt, malignant neuroblastoma for Caspase-3, PARP1 and cleaved-PARP1, and a glioblastoma sample with no mutations for PTEN. A primary human glioblastoma with EGFR gene amplification was used as a positive control for the wild type EGFR (EGFRwt).

### 2.5. Evaluation of Immunohistochemical Staining

In all of the samples, only the proliferating areas were evaluated, while necrotic or regressive areas were excluded from analysis. Antigen expression was evaluated according to the intensity (–, +, ++), frequency of positive nuclei/cells (<20%, 20%–50%, >50%), and distribution (focal or diffuse), with a scoring system that used three categories A, B, and C (Table 1).

The Labelling Index (LI) was calculated as the mean of the areas, of at least 1000 cells. Visual analysis was used to identify the areas with the highest frequency of positive nuclei/cytoplasms. These areas were usually 5 HPF with immersion oil, which corresponded to 0.001 mm^2^.

In GBMs only, Caspase-3 and cleaved-PARP1 expression was calculated as the percentage of positive nuclei/cytoplasms after counting all of the tumor sections.

In addition, in GBMs, the Ki-67/MIB.1 LI was calculated also in areas that showed the maximum pStat3 LI *,* and the pStat3 LI was calculated in the areas with the maximum Ki-67/MIB.1 LI.

### 2.6. Protein Extraction and Western Blotting Analysis

Paraffin sections for protein extraction were deparaffinized and homogenized in RIPA buffer with a protease inhibitor cocktail (Sigma Aldrich Co., St. Louis, MO, USA). After quantification of the total protein lysate using the BCA Protein Assay Kit (Pierce Biotechnology, Rockford, IL, USA), 80 *μ*g of total protein was resolved by SDS-PAGE on a 12% gel. The proteins were then transferred to nitrocellulose membranes (Biorad, Hercules, CA, USA). Blots were incubated overnight at 4°C with a rabbit monoclonal antiphospho-Akt (Ser473) antibody at a dilution of 1:1000 (*#*3787, Cell Signaling Technology), and they were then incubated with the appropriate HRP-conjugated secondary antibody (Dako). Protein signals were detected using the ECL detection system (GE Healthcare, Buckinghamshire, UK). A specific anti-*β*-actin antibody (A5441, Sigma Aldrich Co.) was used to normalize sample loading and transfer. The intensity of the bands was quantified by densitometry using the NIH Image J software (RSB, NIMH, Bethesda, MD, USA).

### 2.7. Statistical Analysis

Associations between the variables were evaluated using 2 × 2 contingency tables and the two-tailed Fisher's exact test. Correlation analyses were performed using the Pearson's correlation coefficient. Survival analysis was carried out using the Kaplan-Meier method (SPSS version 15.0, Chicago, IL, USA).

## 3. Results

### 3.1. EGFR Amplification and Immunohistochemistry (Performed Only in GBMs)

Twelve out of 34 GBMs showed EGFR amplification (35.3%), and 22 of the GBMs were immunopositive for EGFRwt (64.7%). All but one of the 12 cases that carried EGFR amplification were immunopositive for EGFRwt (*P* = .239). Out of the 22 cases that did not have EGFR amplification, 11 were immunopositive for EGFRwt (score B + C).

**Figure 1:**
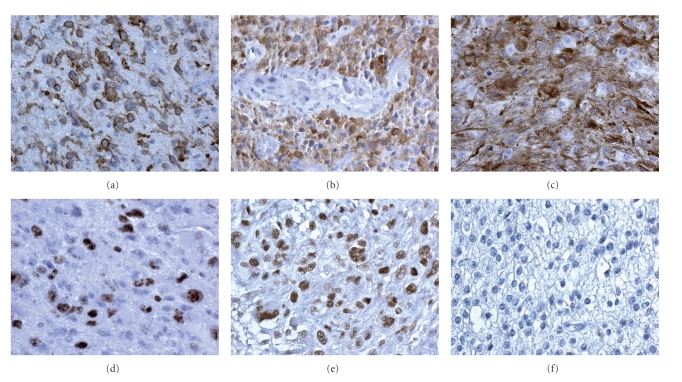
(a) Positive cells for EGFRwt, GBM. (b) Positive cells for EGFRvIII, GBM. (c) PTEN positive staining, GBM. (d) Ki-67/MIB.1 high number of positive nuclei, GBM. (e) pStat3 positive nuclei in GBM. (f) pStat3 negative nuclei in grade II oligodendroglioma. DAB, 400× .

Out of the 12 cases with EGFR amplification, six were positive for EGFRvIII (50%) (*P* = .0403), whereas out of the 22 cases without amplification, only three were positive for EGFRvIII (13.6%) (Table 2). Immunostaining for EGFRwt was either moderate or intense in most of the cells analyzed (Figure 1(a)). Immunostaining for EGFRvIII was moderate, and showed greater regional variability than the EGFRwt immunostaining (Figure 1(b)).

Our cases of glioblastoma were at their first surgery and none derived from a previous astrocytoma. However, a distinction between primary and secondary glioblastoma was not made, also because the only EGFR amplification is not an absolute criterium.

### 3.2. PTEN Mutation Analysis and Immunohistochemistry (Performed Only in GBMs)

All the coding region and the intron-exon boundaries of the PTEN gene were amplified in ten different PCR fragments in the 34 GBM cases.

Direct sequencing led to the identification of four different single nucleotide variations, located in the coding region (*n* = 1) and introns (*n* = 3), respectively. These variations were observed in the four patients resulted negative for PTEN expression by immunohistochemistry.

**Figure 2:**
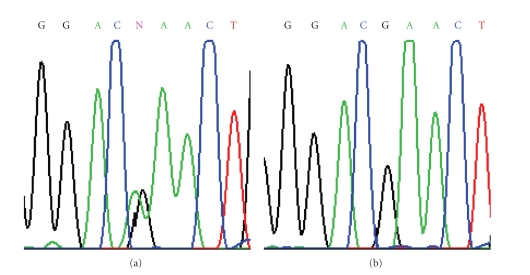
Nucleotide sequence analysis of PTEN exon 5. (a) The nonsynonymous substitution (Arg130Gln, CGA > CAA) from the tumor DNA of Case 6463. (b) The wild-type exon 5 sequence from normal control DNA.

Variation in the coding region is a non-synonymous substitution c.389G → A leading to an amino acid substitution at codon 130 (Arg130Gln), previously confirmed as somatic variant in high-grade gliomas and in gliosarcomas [19, 20]. Among the three intronic variations, one is a new sequence variation, namely IVS8+36C/T; the remaining were IVS8+32T/G and IVS1−97A/G, corresponding to validated SNPs in public database of single nucleotide polymorphisms (http://www.ncbi.nlm.nih.gov/SNP/) (rs555895 and rs1903858, resp.) (Figure 2). The position of the intronic variations is relative to the first (+1) or the last (−1) nucleotide of each intron (Gene Bank sequence NM_000314).

Thirty out of the 34 GBMs were immunopositive for PTEN (88.2%). The staining intensity was strong and uniform in all of the cases. Reactive astrocytes were immunopositive for PTEN, and this staining was also seen in the four tumors that were negative for PTEN immunostaining (Figure 1(c)).

### 3.3. Ki-67/MIB.1

The LI was 5% (2–6%) for grade II astrocytomas, 12% (5–20%) for grade III astrocytomas, 2% (0–10%) for grade II oligodendrogliomas, 15% (12–28%) for grade III oligodendrogliomas, and 23% (12–30%) for GBMs (Figure 1(d)).

### 3.4. Phospho-Stat3 Immunohistochemistry

pStat3 immunostaining was scored as “A” in almost all of the grade II astrocytomas and oligodendrogliomas, as “B” in the grade III astrocytomas and oligodendrogliomas, and as “B” or “C” in GBMs. The pStat3 staining was mainly nuclear, with the exception of few scattered areas where it was cytoplasmic (Figure 1(e)). Cases with positivity <20% were considered as negative. The percentages of positive cells were 0%, 50%, and 56.2% for grade II, III astrocytomas and GBMs, respectively. In oligodendrogliomas, the values were 0% and 20% for grade II and III, respectively (Figure 1(f)).

### 3.5. Phospho-Akt Immunohistochemistry

**Figure 3:**
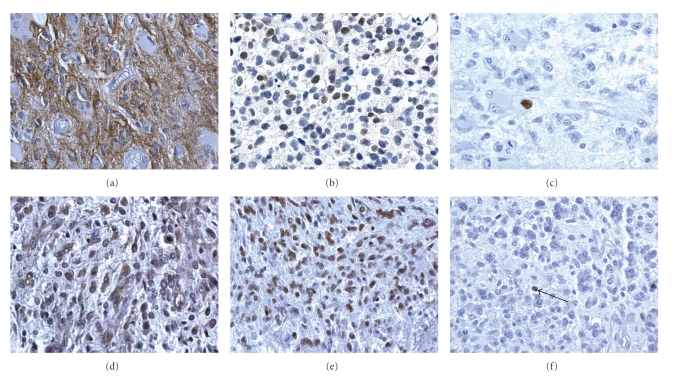
(a) Positive cytoplasmic staining for pAkt, GBM. (b) Positive nuclear staining for pAkt in grade III oligodendroglioma. (c) Caspase-3 positive nucleus, GBM. (d) Beclin 1 positive cytoplasmic and nuclear staining, GBM. (e) PARP1 positive nuclei, GBM. (f) Cleaved-PARP1 positive nucleus, GBM. DAB, 400× .

In grade II astrocytomas and oligodendrogliomas, the staining was mostly nuclear and was scored as “A”. In grade III astrocytomas and oligodendrogliomas, the staining was still nuclear and was scored as “A”. In GBMs, the staining was mainly cytoplasmic, and only occasionally nuclear, and was scored as “C” (Figure 3(a)). In GBMs microvascular proliferations were negative and occasionally the staining was more intense in cells around vessels and outside pseudopalisades. The frequency values were 0%, 20%, and 80% for grade II, III astrocytomas and GBMs, respectively. In oligodendrogliomas, the values were 0% and 20% for grade II and III, respectively (Figure 3(b)).

### 3.6. Phospho-Akt Western Blotting (Performed Only in GBMs)

**Figure 4:**
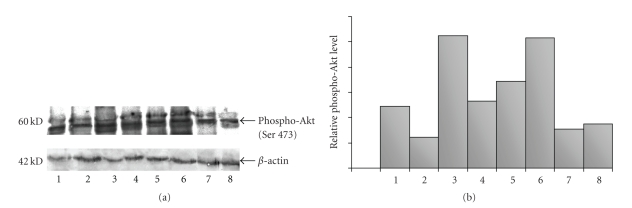
(a) Western blotting of pAkt expression. Positive bands in a sample of 8 cases. (b) Quantitative analysis of pAkt levels normalized to *β*-actin data.

The Western blotting analysis showed variably positive bands (Figures 4(a)-4(b)).

### 3.7. Caspase-3, PARP1, and Cleaved-PARP1 Immunohistochemistry (Performed Only in GBMs)

Caspase-3 was positive as cytoplasmic or nuclear staining or in apoptotic bodies (Figure 3(c)). PARP1 was positive in all the nuclei of the tumor (Figure 3(e)). Cleaved-PARP1 was occasionally positive in nuclei and distributed as Caspase-3 (Figure 3(f)). They were very rare in proliferating areas and more abundant in perinecrotic palisades, which were not counted in this work. The percentages of positive cell/nuclei were constantly <0.02.

### 3.8. Beclin 1 Immunohistochemistry (Performed Only in GBMs)

Beclin 1 was positive in two cases only. The staining was both nuclear and cytoplasmic (Figure 3(d)).

### 3.9. Correlation Analysis

pStat3 and pAkt LIs correlated with the three histological grades (Table 3), but not with survival or with Ki-67/MIB.1 LI within the glioblastoma category. A correlation of pStat3 LI with Ki-67/MIB.1 LI was not found either comparing the mean LI values or the frequency peaks. Also pAkt LI did not correlate with Ki-67/MIB.1 LI. There was a correlation between EGFR amplification status and EGFRwt and EGFRvIII immunohistochemistry (*P* = .0239 and *P* = .0403, resp.), considering scores B + C, but not between EGFRwt and EGFRvIII immunohistochemistry (Table 2). EGFR amplification and immunohistochemistry did not correlate with pStat3. In contrast, correlations were found between EGFRwt immunostaining and levels of pAkt by Western blotting (*P* = .010), between the pAkt levels by Western blotting and immunohistochemistry (*r* = 0.53, *P* = .0348), and the pAkt levels by Western blotting and pStat3 LI by immunohistochemistry (*r* = 0.473, *P* = .0146) (Table 4). The number of cases with positive Beclin 1 was too few, that is, the opposite with the number of cases positive for pStat3, pAkt and Ki-67/MIB.1 LIs in glioblastomas.

The values of Caspase-3 and cleaved-PARP1 LIs were so low as to make the correlation study with the antigens studied be ineffective.

In glioblastomas, no correlation between pStat3, pAkt, Ki-67/MIB.1 LIs and survival was found by Kaplan-Meier analysis.

## 4. Discussion

Demonstration of pStat3 expression in gliomas using immunohistochemistry is not an easy task, especially when attempting to quantitatively evaluate it, due to its heterogeneous expression, which is particularly evident in malignant gliomas. It has been emphasized that this is particularly important in microarray-based studies, since it is crucial that the foci examined are representative of the entire tumor [10]. Heterogeneity in expression can lead to bias when evaluating many antigens in malignant gliomas. There have been attempts to overcome this limitation by the introduction of sophisticated procedures to evaluate positive cells or nuclei when performing immunohistochemical analysis. We used a double-check procedure, and examined antigen expression in full-size tissue sections and in foci with the highest LI values.

In systemic tumors, it has been debated whether pStat3 levels correlate with a worse or a better prognosis, as it has been observed in breast cancer [21]. In our series, pStat3 expression is of moderate intensity, has a nuclear location and its LI significantly increases with malignancy. In glioblastomas, the LI reaches 56.2%, which is less than the values observed in previous studies [7], but is similar to the values that have been reported in more recent studies [11]. As pStat3 is involved in the transformation process, it should have a predictive role for the whole group of astrocytic gliomas, and our results confirm that it does have prognostic significance when comparing the three grades of malignancy. However, pStat3 was not shown to be predictive within the group of malignant gliomas, as it was observed in a previous study [11]. No correlation was found between pStat3 and Ki-67/MIB.1 LI in glioblastomas. The lack of a predictive role for pStat3 in glioblastomas is consistent with observations that many other phenotypic features do not have predictive value. The prognostic role of Ki-67/MIB.1 LI itself in glioblastomas has also been the subject of discussion, and we believe that it is not predictive [22]. Our results are not entirely surprising, since the relationship between pStat3 and proliferation is rather indirect, and is mediated through the inhibition of apoptosis by the activation of the Bcl-2 family [23].

pStat3 inhibition has been shown to have a number of anticancer effects. pStat3 upregulates the transcription of several genes that control tumor cell survival, resistance to apoptosis, cell cycle progression and angiogenesis [3, 5, 6, 23–25], and pStat3 inhibition specifically induces apoptosis [3, 4, 8, 26].

We found a correlation between EGFR amplification and EGFRwt and EGFRvIII immunostaining in our samples. Only one case with an EGFR amplification was negative for EGFR immunohistochemistry, whereas 50% of the cases without EGFR amplification were positive for EGFR immunohistochemistry, and 50% of the cases with EGFR amplification expressed EGFRvIII, in contrast to 13.6% of the cases without amplification. Some of these results are consistent with previous observations [11]. An early hypothesis suggested that pStat3 is activated by deregulation of EGFR and aberrant IL-6 expression [4]. In gliomas, it was observed that the levels of pStat3, together with pAkt, correlate with EGFR aberrations, but specifically with EGFRvIII expression [11]. We did not find any correlation between pStat3 LI and EGFR status. We can only point out that the *P* value of EGFR amplification when compared with pStat3 LI was the lowest of the other nonsignificant comparisons. However, it cannot be ruled out that the small number of samples in our study may have prevented us from finding a potential correlation between pStat3 and EGFR amplification, particularly as pAkt levels correlate with EGFR in a Western blotting analysis. This can be worth also for the lack of correlation between EGFR status and pStat3 by immunohistochemistry. It is to be considered that only four cases of GBMs showed negative PTEN expression, and only one had a real mutation. Anyway, all the four cases without PTEN expression showed already EGFR amplification, with the exception of the only case with a real PTEN mutation.

The impossibility to find any correlation of pStat3 expression with the apoptotic index was due to the short duration of this event and to the too low number of apoptotic nuclei found in proliferating tumor areas, which can be related to the different pathways leading to apoptosis. The action of pStat3, via the Bcl-2 family [5, 23], may lead to the inhibition of transcriptional or intrinsic apoptosis pathway, and may not affect receptor-mediated or extrinsic apoptosis pathway, which may be the major one in perinecrotic tumor areas [27]. The selection of only proliferating areas in our study, and the exclusion of regressive and necrotic ones, may have eliminated the major sources of apoptosis. There is cross-talk between the intrinsic and extrinsic pathway to apoptosis by BH3-interacting domain death agonist (BID) [28]. However, the low detectable values of Caspase-8 in the receptorial pathway to apoptosis in gliomas [29], would make pointless this cross-talk.

Even considering that PTEN was mutated only in one case of our series, no correlation was found between pAkt LI and Ki-67/MIB.1 LI. The levels of pAkt correlate with malignancy within the three grades in our experiments and in other reports [30], but in glioblastomas there is no correlation with the proliferation marker. The explanation can be the same given before for pStat3.

The localization of pStat3 is not so clear-cut in the various observations. It was also shown to be predominantly localized in endothelial cells [31], or in tumor and in endothelial cells [7]. Activated Stat3alpha found in brain tumors was thought to be the result from the action of the endothelial tyrosine kinase VEGFR-2, which plays a central role in autocrine VEGF activation [31]. We found occasional endothelial cells in vascular proliferations of GBMs that stained positive for pStat3, but we could not correlate them with other features of the tumors.

The correlation of pStat3 with pAkt found by us confirms previous observations [11], and emphasizes the position of pStat3 downstream pAkt in the PI3K/Akt signalling pathway [32]. Finally, Beclin 1 was found to be positive in two cases only of glioblastomas, with an inverse correlation with pAkt and pStat3. Beclin 1 is considered to be representative of the type II programmed cell death, or autophagy, and in cancers this pathway is not clearly separated from type I programmed cell death, or apoptosis, and both pathways can be functional in the same cell [33]. In many malignancies, Beclin 1 is monoallelically deleted and is under-expressed. In brain tumors, Beclin 1 levels have been found to decrease with malignancy. In glioblastomas its expression is reduced in comparison with low-grade gliomas, and is rather nuclear indicating a loss of gene function [34]. Our almost negative findings in glioblastomas are consistent with these observations and also with the high expression of pStat3 found in the same cases. In this regard, the induction of autophagy by inhibitors of mTOR [35] and pStat3 [14], in addition to radiation and chemotherapy with temozolomide [36–38], may be significant for therapeutic approaches.

## Figures and Tables

**Table 1:** Scoring system for evaluation of immunostaining.

Marker	Category		
	A	B	C
pStat3 and pAkt	Diffuse, <20%, + with or without foci	Diffuse, 20–50%, ++ with or without foci	Diffuse, >50% or >20%, +++ with multiple foci
Ki-67/MIB.1	<20%	20–30%	>30%
EGFR Amplification	Non-amplified	Non-amplified	More than 2 copies
EGFRwt	−	+/−	+
EGFRvIII	−	+/−	+
PTEN	Positive	—	Negative

Diffuse = homogeneous distribution of positive nuclei. Foci = circumscribed small areas with a higher percentage of positive nuclei. % = number of positive nuclei × 100 nuclei.

**Table 2:** Correlation of EGFR gene amplification status with EGFRwt and EGFRvIII immunohistochemistry in 34 glioblastomas.

EGFR amplification		EGFRwt (B + C) immunohistochemistry		*P* value	EGFRvIII (B + C) immunohistochemistry		*P* value
		Positive (*n* = 22)	Negative (*n* = 12)		Positive (*n* = 9)	Negative (*n* = 25)	
Positive	12 (35.3%)	11 (91.7%)	1 (8.3%)	.0239	6 (50.0%)	6 (50.0%)	.0403
Negative	22 (64.7%)	11 (50.0%)	11 (50.0%)		3 (13.6%)	19 (86.4%)	

**Table 3:** Immunohistochemical frequencies in the three glioma grades.

Activated pathway	Astrocytoma (*n* = 20)		GBM (*n* = 34)	Oligodendroglioma (*n* = 10)	
	Grade II	Grade III	Grade IV	Grade II	Grade III

Stat3	0%	50%	56.2%	0%	20%
Akt	0%	20%	80%	0%	20%

**Table 4:** Correlation of EGFR amplification, EGFRwt and EGFRvIII immunopositivity with pAkt and pStat3 immunohistochemistry.

Activated pathway	EGFR amplification		*P* value
	Positive (*n* = 12)	Negative (*n* = 22)	

Akt IHC	3 (25.0%)	8 (36.4%)	ns
Akt WB*	5 (41.7%)	9 (41%)	ns
Stat3	6 (50.0%)	8 (31.8%)	ns

EGFRwt (B + C) Immunohistochemistry			

	Positive (*n* = 22)	Negative (*n* = 12)	

Akt IHC	7 (31.8%)	4 (33.3%)	ns
Akt WB*	12 (67%)	2 (12%)	.010
Stat3	9 (40.90%)	5 (41.6%)	ns

EGFRvIII (B + C) Immunohistochemistry			

	Positive (*n* = 9)	Negative (*n* = 25)	

Akt IHC	3 (33.3%)	8 (32.0%)	ns
Akt WB*	3 (33%)	11 (44%)	ns
Stat3	5 (55.5%)	9 (36.0%)	ns

ns = not significative. *Only WB values >0.4 were considered.
